# Supplementary material for: Characteristics of antimicrobial stewardship programmes in hospitals of Uganda
Source: PLoS One. 2022 May 10;17(5):e0268032. doi: 10.1371/journal.pone.0268032 (PMC9089898; doi:10.1371/journal.pone.0268032)
Supplement: S1 File — (DOCX) [file pone.0268032.s001.docx]

# Characteristics of antimicrobial stewardship programmes in hospitals of Uganda

**Supplementary information**

**STROBE Statement—Checklist of items that should be included in reports of *cross-sectional studies***

|  | Item No | Recommendation |
| --- | --- | --- |
| **Title and abstract** | 1 | (*a*) Indicate the study’s design with a commonly used term in the title or the abstract  **……………..................................... Page 2, line 9……………………………….....** |
|  |  | (*b*) Provide in the abstract an informative and balanced summary of what was done and what was found  **…………………………………. Page 2, lines 2-36………………………………..** |
| Introduction | | |
| Background/rationale | 2 | Explain the scientific background and rationale for the investigation being reported  **…………………………….. .pages 3-6, lines 38-109…………………………….** |
| Objectives | 3 | State specific objectives, including any prespecified hypotheses  **………………... pages 2, lines 5-7………………pages 6……lines …106-108** |
| Methods | | |
| Study design | 4 | Present key elements of study design early in the paper  **……………………………… page 8, lines 152-158 …………………………………** |
| Setting | 5 | Describe the setting, locations, and relevant dates, including periods of recruitment, exposure, follow-up, and data collection  **………………… pages 7-8, lines 114-159……………………** |
| Participants | 6 | (*a*) Give the eligibility criteria, and the sources and methods of selection of participants  **………………………….. page 9, lines 154-174………………………………….** |
| Variables | 7 | Clearly define all outcomes, exposures, predictors, potential confounders, and effect modifiers. Give diagnostic criteria, if applicable  **…..page 10.….lines 177-192……………………………………..** |
| Data sources/ measurement | 8* | For each variable of interest, give sources of data and details of methods of assessment (measurement). Describe comparability of assessment methods if there is more than one group  **………………… page 10, lines 177-192………………………………………** |
| Bias | 9 | Describe any efforts to address potential sources of bias  **………………………. Page 9, lines 161-172…………………………….** |
| Study size | 10 | Explain how the study size was arrived at  **……………… page 9, lines 161-174……………..** |
| Quantitative variables | 11 | Explain how quantitative variables were handled in the analyses. If applicable, describe which groupings were chosen and why  **…………………… page 13, lines 250-256…………………………….** |
| Statistical methods | 12 | (*a*) Describe all statistical methods, including those used to control for confounding  **………………..pages 13, lines 250-256………………………………..** |
|  |  | (*b*) Describe any methods used to examine subgroups and interactions  **…………………not applicable………………………………….** |
|  |  | (*c*) Explain how missing data were addressed  **…………………..page12-13, lines 240-246………………………………..** |
|  |  | (*d*) If applicable, describe analytical methods taking account of sampling strategy  **………………….page 13 lines 254-255……………………………………** |
|  |  | (*e*) Describe any sensitivity analyses  **…………………………Not applicable………………………………………..** |
| Results | | |
| Participants | 13* | (a) Report numbers of individuals at each stage of study—eg numbers potentially eligible, examined for eligibility, confirmed eligible, included in the study, completing follow-up, and analysed  **………………..page 14 lines 261-270 …………………………** |
|  |  | (b) Give reasons for non-participation at each stage  **………….page 9, line 173-175……………** |
|  |  | (c) Consider use of a flow diagram  **………** (Appendix S5 ). **……….** |
| Descriptive data | 14* | (a) Give characteristics of study participants (eg demographic, clinical, social) and information on exposures and potential confounders  **…………………page 11, lines 262-273……………** |
|  |  | (b) Indicate number of participants with missing data for each variable of interest  **………………………not applicable………………………………………………..** |
| Outcome data | 15* | Report numbers of outcome events or summary measures  **………………………page 15, lines 174-180………Pages 17, lines 286-308……Pages 21 lines 312-317………………..** |
| Main results | 16 | (*a*) Give unadjusted estimates and, if applicable, confounder-adjusted estimates and their precision (eg, 95% confidence interval). Make clear which confounders were adjusted for and why they were included  **………………………Not applicable ………………………….** |
|  |  | (*b*) Report category boundaries when continuous variables were categorized  **………………………………Page 13 ……lines 253…………………………** |
|  |  | (*c*) If relevant, consider translating estimates of relative risk into absolute risk for a meaningful time period  **………………………not applicable …………………………….** |
| Other analyses | 17 | Report other analyses done—eg analyses of subgroups and interactions, and sensitivity analyses  **…………………………… None …………………………………….** |
| Discussion | | |
| Key results | 18 | Summarise key results with reference to study objectives  **……..pages 23-27, lines 333-352,356-399,402-410………………** |
| Limitations | 19 | Discuss limitations of the study, taking into account sources of potential bias or imprecision. Discuss both direction and magnitude of any potential bias  **……...............page17, lines 313-324………………………….** |
| Interpretation | 20 | Give a cautious overall interpretation of results considering objectives, limitations, multiplicity of analyses, results from similar studies, and other relevant evidence  **……………..pages 27-28, lines 412-428……………………..** |
| Generalisability | 21 | Discuss the generalisability (external validity) of the study results  **…Pages 9, .lines 160-175……………page 28, lines 424 -428………………………..** |
| Other information | | |
| Funding | 22 | Give the source of funding and the role of the funders for the present study and, if applicable, for the original study on which the present article is based  **……should not appear in the manuscript as per the guidelines of PLoS ONE……** |

*Give information separately for exposed and unexposed groups.

**Note:** An Explanation and Elaboration article discusses each checklist item and gives methodological background and published examples of transparent reporting. The STROBE checklist is best used in conjunction with this article (freely available on the Web sites of PLoS Medicine at http://www.plosmedicine.org/, Annals of Internal Medicine at http://www.annals.org/, and Epidemiology at http://www.epidem.com/). Information on the STROBE Initiative is available at www.strobe-statement.org.
