# Supplementary material for: Characteristics of antimicrobial stewardship programmes in hospitals of Uganda
Source: PLoS One. 2022 May 10;17(5):e0268032. doi: 10.1371/journal.pone.0268032 (PMC9089898; doi:10.1371/journal.pone.0268032)
Supplement: S2 File — (DOCX) [file pone.0268032.s002.docx]

# CHARACTERISTICS OF ANTIMICROBIAL STEWARDSHIP PROGRAMMES IN HOSPITALS OF UGANDA

# Supplementary information

# Informed consent for the above study

Give the names and institutions of the investigators.

1. **Isaac Kimbowa** (*BSc.Pharm, PGD. ACREM, PGD.PPM, MSc Pharm, MBA*)

Department of Pharmacology of Therapeutics, School of Biomedical Sciences, College of Health Sciences, Makerere University .Room 10.+256-786403721/075747798.Email: [jakemagulu@gmail.com](mailto:jakemagulu@gmail.com)

1. **Assoc Prof Joan Kalyango (B. Pharm, MSc, PhD)**

Department of Clinical epidemiology, School of Medicine, College of Health Sciences, Makerere University

Email: [nakayaga2001@yahoo.com](mailto:nakayaga2001@yahoo.com), Contacts: 256-772412548

1. **Dr Ocan Moses (BSc. Pharm, PGD-ACREM, MSc, PhD)**

Department of Pharmacology and Therapeutics, School of Biomedical Sciences,

College of Health Sciences, Makerere University

Email: [ocanmoses@gmail.com](mailto:ocanmoses@gmail.com), Contacts: +256-751828420/

1. **Prof Cecilia Stalsby Lundborg**

Department of Public Health / Karolinska institutet, 17177 Stockholm, Sweden.

Email: [Cecilia.stalsby.Lundborg@ki.se](mailto:Cecilia.stalsby.Lundborg@ki.se),Contacts: +46(0)852483366/+46(0)702102673

**Background and rationale for the sub-study I:**

Give a brief background and rationale for the proposed research.

Antimicrobial stewardship (AMS) is the optimal selection of antibacterials agent in right formulation, dose, frequency, and duration to generate the best clinical outcome with minimal toxicity and negligible impact on resistance development. Evidence of Antimicrobial stewardship practices has been majorly derived from adult data in high-income countries. There is a scarcity of data on ASM practices in Low-Middle- Income countries like Uganda. Regional referral hospitals, General hospitals are challenged with the growing burden of antibacterial resistances and most studies conducted in these health facilities have recommended the establishment or strengthening of Antimicrobial stewardship practices. However, in this facilities implementation of ASM practices are challenged by a lack of supporting policies, structures, human resources. Several initiatives have been played to introduce AMS in health facilities, their implementation, outcomes, and challenges have remained unknown.

**Purpose:**

**Brief description of the purpose of the study and why the participant is being asked to participate. A statement that the study involves experimentation and what part of the study is experimental.**

This study aims to determine the Antimicrobial stewardship programmes, actices being used to optimize antibacterial use in selected health facilities of Uganda. The study will administer a structured interview to heads of infection prevention and control who could come from any of the known departments of the hospitals such as Paediatrics, Outpatients, Pharmacy, Emergency, Surgery, Obstetrics and Gynaecology, Private wing, Medicines and Therapeutic committee, Infection control committee, Medical Director. These have been selected because they are the stewards or head and influence practice and prescribe and administer antibacterial agents to children.

**Who will participate in the study?**

The study will interview heads of infection control in regional referral , general hospitals in nine and PNFPs. Each interview will take 45minutes to one hour with the key informant.

**Risks/Discomforts:**

Description of the possible risks and discomforts a participant might experience while in the study.

The study will administer a questionnaire with questions expected to confer a low risk of physical harm or psychological discomfort. In administering the questionnaire, there is minimal chance of discussing issues that cause some level of discomfort.

**Benefits:**

Anticipated benefits of conducting the study including possible benefits to the participant, community and the entire scientific world.

The study may confer indirect benefits to the respondents, such as self-acknowledgement and validation of the AMS practices in different wards or units. It may increase self-awareness as well as a sense of empowerment in all AMS practices.

**Confidentiality:**

Explanation of how privacy will be maintained during the study and how confidential and sensitive personal information to the participant will be handled. Please mention who you expect to have access to confidential information.

The information collected in this study will be kept confidential. Information about you that will be collected during the research will be put away, and no-one except the researchers will access it. Any information about you will have a number on it instead of your name. Only the researchers will know your number and be kept under a lock and key cupboard. It will not be shared with or given to anyone except supervisors who will have access to the information, such as research sponsors, HDREC.

**Alternatives:**

Participants should be informed that participation in the study is not mandatory and possible alternatives other than participating in the study.

Your participation in this research is entirely voluntary. It is your choice whether to participate or not. Whether you choose to participate or not, nothing will happen concerning your work or activities. It your right to change your mind during or before the study or stop participating even if you agreed earlier.

**Cost:**

The possible costs to be met during the study's conduct as far as the particular participant is concerned.

Your participation is free. There will be no money given to you to induce to participate or gifts to take part in this research

**Compensation for participation in the study:**

Explain if participants will be compensated for participating in the study and how they will be compensated. Also explain what happens if a participant is injured during their course of participation and how they will be treated. State how participants who suffer permanent damage will be compensated.

Since this is a questionnaire or interview study with minimal harm, we shall have minimal physical damage to participants. Risks of permanent damage are minimal in such study.

**Reimbursement:**

State how participant costs in terms of travel and opportunity cost while they come to the study site will be met.

Since the interview or questionnaire will take one hour or 45 minutes. An incentive of 20,000/= will be prepared in case someone is met at another location where he incurs costs of transport.

**Questions:**

State how participants who have study related questions can reach investigators to answer such questions.

This research has been reviewed and approved by the School of Biomedical Sciences Higher Degree Ethics and Research Ethics Committee.

**If you have any further questions or concerns about this study, please contact:**

**Name of researcher**: Kimbowa Isaac

**Full address**: Makerere University College of Health Sciences, School of Biomedical Sciences, Department of Pharmacology and Therapeutics, room 10.P.O.BOX 7072, Kampala .**Tel:** 0786403721/0757747798

**E-mail**: jakemagulu@gmail.com

You can also contact (Researchers name) supervisor:

Name of the researcher; Assoc **Prof Joan Kalyango**

Full address: **Department of Pharmacy, School of Health Sciences, College of Health Sciences**

Tel: 256-772412548 E-mail: [nakayaga2001@yahoo.com](mailto:nakayaga2001@yahoo.com),

**Questions about participants rights:**

Explain how participants who have questions about their rights as research participants can have their queries addressed.

**If you have questions concerning your rights or welfare, please contact** Chairperson School of Biomedical Sciences Higher Degree Ethics and Research Ethics Committee (HDREC).

**HDREC Chairperson name**: Dr Mwaka Erisa

**Full address**: Makerere University College of Health Sciences, School of Biomedical Sciences, Department of Anatomy. P.O.BOX 7072, Kampala. Tel**:** 0752575050

**E-mail**: erisamwaka@yahoo.com

**Informed Consent**

I have read, and I understand the contents of the information sheet and understand that I have been invited to participate, that my participation is voluntary. I understand that I can withdraw my participation at any time in this study.

**__ISAAC KIMBOWA_____**

Printed Researcher Name

**_____________________________________Date ____________________**

Researchers Signature Date

**Consent given by**

**_____________________________________Date ____________________**

Respondents Signature Date
