# Supplementary material for: Characteristics of antimicrobial stewardship programmes in hospitals of Uganda
Source: PLoS One. 2022 May 10;17(5):e0268032. doi: 10.1371/journal.pone.0268032 (PMC9089898; doi:10.1371/journal.pone.0268032)
Supplement: Attachment — Submitted filename: PONE-D-21-34980_reviewer.pdf [file pone.0268032.s004.pdf]

PLOS ONE

CHARACTERISTICS OF ANTIMICROBIAL STEWARDSHIP PROGRAMMES IN HOSPITALS OF UGANDA

--Manuscript Draft--

**Manuscript Number:**	PONE-D-21-34980
**Article Type:**	Research Article
**Full Title:**	CHARACTERISTICS OF ANTIMICROBIAL STEWARDSHIP PROGRAMMES IN HOSPITALS OF UGANDA
**Short Title:**	Antimicrobial stewardship programmes in hospitals of Uganda
**Corresponding Author:**	Isaac Magulu Kimbowa, MSc, MBA<br>Makerere University College of Health Sciences<br>Kampala, Mulago UGANDA
**Keywords:**	Keywords: Antimicrobial stewardship, antimicrobial stewardship programmes, antimicrobial stewardship strategies
**Abstract:**	While interest in antimicrobial stewardship programmes (ASP) is growing in most low- and middle-income countries (LMICs), there is a paucity of information on their adoption or implementation in Africa, particularly Uganda. In our study, we assessed the characteristics of ASPs, implemented AMS strategies and the challenges to their implementation in hospitals in Uganda. We conducted a cross-sectional study among heads of infection prevention committees (IPCs) in regional referral hospitals, general hospitals, and private-not-for-profit (PNFP) hospitals from October 2019 to February 2020. An interview administered questionnaire was used to collect data on the presence of ASP in the studied hospitals, the characteristics of ASP, the implementation of the AMS strategies, and the challenges of implementing ASP. We analysed all data using descriptive statistics. A total of 32 heads of infection prevention committees of the study hospitals were enrolled on the study. Of these, eight (08) were from regional referral hospitals, twenty-one (21) were from the general hospitals, and three (03) were from private-not-for profit (PNFPs). Most heads of IPC were pharmacists (17/32, 53.1%) with a mean age and standard deviation (sd) of 36.1 (+ 1.1) years. A formal ASP was adopted or implemented in 14 out of the 32 (44%, 95% CI 26-62) studied hospitals. Thirty out of 32 hospitals implemented at least one type of AMS strategy. Sixty-eight percent (22/32) hospitals implemented pre-authorisation and approval as their primary AMS core strategy in optimising antibacterial use. The most commonly reported challenges to the implementation of ASP across all 32 hospitals (with or without ASP) were a lack of time for the ASP team (29/32, 90.6%) and a lack of allocated funding for the antimicrobial stewardship team (29/32, 90.6%). In this study, most hospitals in Uganda implemented at least one AMS strategy despite the low implementation of ASP in hospitals. The ministry of health needs to sensitise and support the establishment of ASP in hospitals across the country.
**Order of Authors:**	Isaac Magulu Kimbowa, MSc, MBA
	Moses Ocan, PhD
	Jaran Eriksen, PhD
	Mary Nakafeero, MSc
	Celestino Obua, PhD
	Cecilia Stalsby.Lundborg, PhD
	Joan Kalyango, PhD
**Additional Information:**	
**Question**	**Response**
**Financial Disclosure**<br><br>Enter a financial disclosure statement that	No-The funders had no role in study design, data collection and analysis, decision to publish, or preparation of the manuscript.

Powered by Editorial Manager® and ProduXion Manager® from Aries Systems Corporation

describes the sources of funding for the work included in this submission. Review the submission guidelines for detailed requirements. View published research articles from *PLOS ONE* for specific examples.<br><br>This statement is required for submission and **will appear in the published article** if the submission is accepted. Please make sure it is accurate.<br><br>**Unfunded studies**<br>Enter: *The author(s) received no specific funding for this work.*<br><br>**Funded studies**<br>Enter a statement with the following details:<br>• Initials of the authors who received each award<br>• Grant numbers awarded to each author<br>• The full name of each funder<br>• URL of each funder website<br>• Did the sponsors or funders play any role in the study design, data collection and analysis, decision to publish, or preparation of the manuscript?<br>• **NO** - Include this sentence at the end of your statement: *The funders had no role in study design, data collection and analysis, decision to publish, or preparation of the manuscript.*<br>• **YES** - Specify the role(s) played.<br><br>* typeset	
**Competing Interests**<br><br>Use the instructions below to enter a competing interest statement for this submission. On behalf of all authors, disclose any competing interests that could be perceived to bias this work—acknowledging all financial support and any other relevant financial or non-financial competing interests.<br><br>This statement is required for submission and **will appear in the published article** if the submission is accepted. Please make sure it is accurate and that any funding	The authors have declared that no competing interests exist.

<table>
<tr>
<td>sources listed in your Funding Information later in the submission form are also declared in your Financial Disclosure statement.

View published research articles from PLOS ONE for specific examples.

NO authors have competing interests

Enter: The authors have declared that no competing interests exist.

Authors with competing interests

Enter competing interest details beginning with this statement:

I have read the journal's policy and the authors of this manuscript have the following competing interests: [insert competing interests here]

* typeset</td>
<td></td>
</tr>
<tr>
<td>Ethics Statement

Enter an ethics statement for this submission. This statement is required if the study involved:

• Human participants
• Human specimens or tissue
• Vertebrate animals or cephalopods
• Vertebrate embryos or tissues
• Field research

Write "N/A" if the submission does not require an ethics statement.

General guidance is provided below. Consult the submission guidelines for detailed instructions. Make sure that all information entered here is included in the Methods section of the manuscript.</td>
<td>The Makerere University School of Biomedical Sciences Higher Degree Research and Ethics Committee approved our study procedure (SBH-HDREC-624). The Uganda National Council of Science and Technology (UNCST) granted further ethical approval for the project (HS339ES). All respondents involved in this study provided written informed consent. A consent form was included in the questionnaire, outlining the study goal and ensuring respondents' privacy and confidentiality, as well as the voluntary nature of their participation.</td>
</tr>
</table>

Powered by Editorial Manager® and ProduXion Manager® from Aries Systems Corporation

**Format for specific study types**<br><br>**Human Subject Research (involving human participants and/or tissue)**<br>• Give the name of the institutional review board or ethics committee that approved the study<br>• Include the approval number and/or a statement indicating approval of this research<br>• Indicate the form of consent obtained (written/oral) or the reason that consent was not obtained (e.g. the data were analyzed anonymously)<br><br>**Animal Research (involving vertebrate animals, embryos or tissues)**<br>• Provide the name of the Institutional Animal Care and Use Committee (IACUC) or other relevant ethics board that reviewed the study protocol, and indicate whether they approved this research or granted a formal waiver of ethical approval<br>• Include an approval number if one was obtained<br>• If the study involved *non-human primates*, add *additional details* about animal welfare and steps taken to ameliorate suffering<br>• If anesthesia, euthanasia, or any kind of animal sacrifice is part of the study, include briefly which substances and/or methods were applied<br><br>**Field Research**<br><br>Include the following details if this study involves the collection of plant, animal, or other materials from a natural setting:<br>• Field permit number<br>• Name of the institution or relevant body that granted permission	
**Data Availability**<br><br>Authors are required to make all data underlying the findings described fully available, without restriction, and from the time of publication. PLOS allows rare exceptions to address legal and ethical concerns. See the PLOS Data Policy and FAQ for detailed information.	Yes - all data are fully available without restriction

A Data Availability Statement describing where the data can be found is required at submission. Your answers to this question constitute the Data Availability Statement and **will be published in the article**, if accepted.<br><br>**Important:** Stating 'data available on request from the author' is not sufficient. If your data are only available upon request, select 'No' for the first question and explain your exceptional situation in the text box.<br><br>Do the authors confirm that all data underlying the findings described in their manuscript are fully available without restriction?	
**Describe where the data may be found in full sentences. If you are copying our sample text, replace any instances of XXX with the appropriate details.**<br><br>• If the data are **held or will be held in a public repository**, include URLs, accession numbers or DOIs. If this information will only be available after acceptance, indicate this by ticking the box below. For example: *All XXX files are available from the XXX database (accession number(s) XXX, XXX.)*.<br>• If the data are all contained **within the manuscript and/or Supporting Information files**, enter the following: *All relevant data are within the manuscript and its Supporting Information files.*<br>• If neither of these applies but you are able to provide **details of access elsewhere**, with or without limitations, please do so. For example:<br><br>*Data cannot be shared publicly because of [XXX]. Data are available from the XXX Institutional Data Access / Ethics Committee (contact via XXX) for researchers who meet the criteria for access to confidential data.*<br><br>*The data underlying the results presented in the study are available from (include the name of the third party*	The data is held in a public repository called open science framework (OSF). Its DOI 10.17605/OSF.IO/8TNBZ.<br>The link to all supporting documents is<br>https://osf.io/8tnbz/?view_only=e3459dfe896b44a3902f65e48ec2bd44

and contact information or URL).<br>• This text is appropriate if the data are owned by a third party and authors do not have permission to share the data.<br><br>* typeset	
Additional data availability information:	Tick here if the URLs/accession numbers/DOIs will be available only after acceptance of the manuscript for publication so that we can ensure their inclusion before publication.

Powered by Editorial Manager® and ProduXion Manager® from Aries Systems Corporation

Manuscript Click here to access/download;Manuscript;manuscript .docx

CHARACTERISTICS OF ANTIMICROBIAL STEWARDSHIP PROGRAMMES IN HOSPITALS OF UGANDA

Isaac Kimbowa[1*], Moses Ocan[2,] Jaran Eriksen[3,4], Mary Nakafeero[5,] Celestino Obua[6], Cecilia Stålsby Lundborg[7], Joan Kalyango[8,9]

1; Department of Pharmacology and Therapeutics, Makerere University College of Health Sciences, Kampala, Uganda

2; Department of Pharmacology and Therapeutics, Makerere University College of Health Sciences, Kampala, Uganda

3; Department of Global Public Health, Karolinska Institutet, Stockholm, Sweden

4; Unit of Infectious diseases/Venhälsan, Stockholm South Hospital, Stockholm, Sweden

5; School of Public Health, Makerere University College of Health Sciences, Kampala, Uganda,

6; Mbarara University of Science and Technology, Mbarara, Uganda,

7; Department of Global Public Health, Karolinska Institutet, Stockholm, Sweden

8; Clinical Epidemiology Unit, Makerere University College of Health Sciences, Kampala, Uganda

9; Department of Pharmacy, Makerere University College of Health Sciences, Kampala, Uganda

* jakemagulu@gmail.com (IK.)

Keywords: *Antimicrobial stewardship, antimicrobial stewardship programmes, antimicrobial stewardship strategies*

Abstract

While interest in antimicrobial stewardship programmes (ASP) is growing in most low- and middle-income countries (LMICs), there is a paucity of information on their adoption or implementation in Africa, particularly Uganda. In our study, we assessed the characteristics of ASPs, implemented AMS strategies and the challenges to their implementation in hospitals in Uganda. We conducted a cross-sectional study among heads of infection prevention committees (IPCs) in regional referral hospitals, general hospitals, and private-not-for-profit (PNFP) hospitals from October 2019 to February 2020. An interview administered questionnaire was used to collect data on the presence of ASP in the studied hospitals, the characteristics of ASP, the implementation of the AMS strategies, and the challenges of implementing ASP. We analysed all data using descriptive statistics. A total of 32 heads of infection prevention committees of the study hospitals were enrolled on the study. Of these, eight (08) were from regional referral hospitals, twenty-one (21) were from the general hospitals, and three (03) were from private-not-for profit (PNFPs). Most heads of IPC were pharmacists (17/32, 53.1%) with a mean age and standard deviation (sd) of 36.1 (±1.1) years. A formal ASP was adopted or implemented in 14 out of the 32 (44%, 95% CI 26-62) studied hospitals. Thirty out of 32 hospitals implemented at least one type of AMS strategy. Sixty-eight percent (22/32) hospitals implemented pre-authorisation and approval as their primary AMS core strategy in optimising antibacterial use. The most commonly reported challenges to the implementation of ASP across all 32 hospitals (with or without ASP) were a lack of time for the ASP team (29/32, 90.6%) and a lack of allocated funding for the antimicrobial stewardship team (29/32, 90.6%). In this study, most hospitals in Uganda implemented at least one AMS strategy despite the low implementation of ASP in hospitals. The ministry of health needs to sensitise and support the establishment of ASP in hospitals across the country.

Introduction

Antimicrobial stewardship programmes (ASPs) are multidisciplinary programmes that optimise antibacterial use to improve patient outcomes and safety, thus reducing healthcare-related costs and reducing the development of antimicrobial resistance (AMR) [1-3]. It is critical to optimise currently used antibacterial agents in communities and hospitals to conserve their efficacy whilst reducing the development of antimicrobial resistance [4]. The strategies on optimisation of antibacterial use have been on the national and international agenda, where numerous professional organisations, including clinical and public health and regulatory and accreditation agencies, have advocated for implementing antimicrobial stewardship (AMS) interventions to curb the growing burden of antimicrobial resistance (AMR) [2, 5, 6]. Antimicrobial stewardship (AMS) is a set of strategies, policies, guidelines, or tools that optimise antibacterial use and reduce AMR development [7, 8].

A decade after coining the term "antimicrobial stewardship," the Infectious Diseases Society of America (IDSA) and the Society for Healthcare Epidemiology of America (SHEA) released the first guidelines in 2007 for establishing ASPs in hospitals.[7, 9-11]. The guidelines formed a blueprint for developing ASP structure, leadership, and governance and laid a procedure for selecting AMS core and supplemental strategies for different hospitals [7, 8, 12]. The guidelines recommended implementing three primary AMS core strategies: pre-authorisation, antimicrobial formulary restriction, post prescription audit, and review. These three core strategies were used to characterise ASP in a given hospital depending on their implemented strategy [13]. Post prescription audit and review has been reported in multiple studies to impose a low burden on prescribers' prescription autonomy and not prevent the prompt initiation of antibacterial therapy [12]. The implementation of these primary core AMS strategies and their scalability depends on the capacity of the ASPs [3, 14].

Furthermore, the recommendations advocated for supplemental AMS strategies (education, guidelines, clinical pathways, order forms, dosage optimisation, and de-escalation) to support optimisation of antibacterial use[10, 12]. The choice of a particular strategy depends on resources and expert availability in the hospitals [8, 15]. Several studies have shown that intervention AMS strategies vary amongst hospital ASPs, with the number and type of personnel involved in the programme influencing the type of strategies, whose choice depended on resources and expert availability in the hospitals [8, 15].

Over the last two decades, hospital ASPs have helped reduce antibacterial consumption and improve the selection of antibacterials [16, 17]. The implementation of the programmes in communities have also increased public awareness of antibacterial use, minimised overuse in animals and human, improved clinical outcomes and reduced the burden of AMR [6, 12]. The emergency of the first guidelines on ASP from IDSA supported the development of regulatory frameworks on ASP and their implementation in every hospital that needed accreditation in high-income countries than in low-and middle-income countries (LMICs) [7, 18]. However, to support the development of ASPs in LMICs, the World Health Organisation (WHO) developed a Global Action Plan (GAP) on AMR. The GAP on AMR emphasised ASPs implementation in all member countries' human and animal sectors as a key intervention to combat AMR through optimising antibacterial use [19]. Additionally, they also developed a tool kit to guide the adoption or development of ASPs structure, core elements, procedures and strategies at the hospital and country-level among LMICs [20]. Over two-thirds of the member nations of WHO who endorsed the GAP on AMR have implemented national action plans on antimicrobial resistance, emphasising ASP implementation in hospitals after the endorsement of GAP on AMR [21].

However, despite the growing interest in adopting or implementing ASP in LMICs, there is still slow adoption of ASPs in hospitals [22-27]. Information regarding the availability and characteristics of ASPs in Uganda hospitals remains unknown despite the capacity development of healthcare professionals and ongoing implementation of NAP on AMR [28, 29]. Therefore, the study aimed to determine the characteristics of antimicrobial stewardship programmes (ASP), implemented AMS strategies and the challenges to their implementation in regional referral hospitals (RRH), general (district) hospitals and tertiary PNFPs in Uganda. The findings will offer policymakers in the Ministry of Health the information required to implement ASP in hospitals in Uganda.

MATERIAL AND METHODS

Study setting

We conducted this study at regional referral, general hospitals and tertiary private not for profit hospitals (PNFPs) for six weeks between November 2019 to February 2020 in Uganda (supplementary information 5 of a map of study setting). Over 45% health facilities are public, 40% are private not for profit (PNFPs) and 15% private for-profit (PFP) [30, 31]. Public health facilities are hierarchical in terms of referral and do offer free health service delivery. They have comprised Health Center I, II, III, and IV, followed by General Hospitals (GH), Regional Referrals hospitals (RRH), and National Referral Hospitals (NRH). The PNFP offer subsidised service compared to PFP since they receive government funding [30]. Four PNFP hospitals offer specialised services to the urban population, while 40 general hospitals PNFPs serve communities.

Recently, healthcare professionals from public health facilities, PNFPs and communities were trained to implement AMS strategies to optimise antibacterial use in human and animal sectors[28]. Furthermore, the ministry of health established medicines and therapeutics committees (MTCs) and infection prevention committees (IPC) health as systems strengthening exercises to improve medication use in higher public health facilities and PNFPs[29]. To date, there is limited information on characteristics of ASP in regional referral, general hospitals and PNFP. These three types of hospitals (regional referral, general hospitals and PNFPs) were selected because they are expected to develop and implement ASPs and implementing AMS strategies after numerous capacity-building initiatives[28].

Study design and participants

We conducted a cross-sectional study in regional referrals, general hospitals and PNFPs. The study respondents included heads or chairs of infection prevention committees (IPCs) purposively selected because their expertise and experience spanned from administration, research, infection control and prevention, medicines and therapeutic committee participation. Their position would allow them to respond appropriately, thus minimising confounding and bias of the outcome since they are privy to any matter, including antimicrobial stewardship, because they attend different hospitals' committees and administration meetings. After receiving administrative clearance from each hospital, the study used various communication strategies (emails, phone calls, and personal visits) to increase IPC heads' participation.

Sampling procedure

We employed a three-stage sampling procedure to select the public hospitals in our study. In the first stage, we selected two regional referral facilities per region using simple random sampling (lottery techniques) from each pool of regional facilities (central (5), north (4), West (4), and East (3) sampling frames. For this study, we chose eight regional referral hospitals out of 16 (50%) from all four regions. In the second stage, we used a two-criterion system to select general hospitals referring patients to the already selected regional referral in the first stage. Secondly, the general hospital selected had to be within one hour by ambulance drive to the already selected regional referral hospital to abide by the referral pattern of patients. We selected 32 out of 47 (68%) general hospitals. We randomly selected three out of four (75%) tertiary PNFPs graded at a regional referral health facility level.

In the third stage, we purposively selected heads of infection prevention committees hospitals (study regional referral hospitals, general hospitals and tertiary PNFP. that granted administrative clearance in each of the

Variables

The outcome variables of this study were the proportion of antimicrobial stewardship programmes, AMS strategies and challenges of implementation of programmes in health facilities. In each health facility, the head of the infection prevention committee was asked whether they had a formal ASP programme. The response to the question was "yes" or "no". However, the study tool never provided a standard definition of an ASP because no formal guidelines or policies require any hospital to develop ASP. However, hospitals were classified as having an ASP after each of the heads of the infection control committee of the given hospital responded yes on the first cardinal question of whether they had an ASP or not after. AMS strategies were into two (policy items), three (core items),

and 14 (supplementary items) between hospitals with ASP and those without ASP. Core strategies were defined as mandatory strategies adopted or implemented in the ASP, and these are abided by all hospital departments. Policy AMS strategies were defined as strategies adopted from national policy and implemented by the health facility to improve antibiotic use in the hospitals. Supplementary strategies are optional strategies that can be adopted on top of core strategies to optimise antibacterial use in health facilities. The item statements for AMS Strategies were measured using yes or no. The challenges of implementing an ASP had 19 item statements to which responses were "yes" or "no". We assessed challenges to implementing an ASP in hospitals with existing programmes and then assessed challenges in those without a programme and the responses. Their challenges were assessed responses of "yes" or "no". Independent variables included hospital characteristics such as the type of health facility (general, regional referral, and private-not for-profit), teaching status (teaching and non-teaching hospital) and bed capacity.

Questionnaire development

The questionnaire (supplementary information file 3) was developed in consultation with experts on infectious diseases, epidemiology, microbiology and pharmacology, public health. It was developed in English after searching the literature on previous similar studies on ASP, AMS strategies and challenges [3, 10, 12, 20]. We used workshops and meetings to enable experts to agree on the sections of the draft questionnaire, item or number of questions, comprehensiveness and clarity of the questions in every section, and item relevance in the questionnaire before the questionnaire was pre-tested. The questionnaire was pre-testing among twenty healthcare providers who were members of

the infection control committee to check for clarity and comprehension. Cronbach alpha for AMS strategies was 0.8516, while for challenges of implementing ASP was 0.7716.

Data collection

We collected data from heads of infection prevention committees using an interviewer-administered questionnaire. Research assistants such as pharmacists, medical officers, nurses, and hospital statisticians were trained on the questionnaire before data collection. We informed every participant that their participation was voluntary. After accepting to participate, the research assistant provided a brief introduction of the study, objective, and procedures and informed the respondent of their voluntary participation, anonymity, and confidentiality declarations. The survey was voluntary, and no incentives were given, and no individual participant identifiers were collected.

The following sections of the questionnaire were used to gather data: hospital characteristics (section A), demographic and general information (section B), antimicrobial stewardship programmes and AMS strategies (section F), and challenges of implementing ASP (section G) (section I). During data collection, the first question asked of participants was whether the hospital had a formal ASP (formally approved and recognised by the hospital administration). More questions were asked about the AMS strategies (policy, core and supplemental) stewardship strategies performed within the facility. We asked them about the challenges they faced in implementing the ASP.

Data processing and management

The research assistant evaluated every questionnaire for accuracy and completeness at the end of each day's data collection. During fieldwork and data cleaning, we performed a thorough case analysis to detect missing data on variables in the questionnaires. We dropped any questionnaires containing

significant missing data in their variables during the data cleaning process. We utilised EpiDATA manager version 4.2 to conduct double data entry and validation, during which data collection tools were entered twice by different data entrants, which we reconciled to detect any differences or discrepancies. We performed data validation during data entry to reconcile any files that had deviated from the original files.

Data analysis

Data were analysed using STATA 15.1 (StataCorp, Texas, USA). We summarised categorical variables using frequencies and proportions, while continuous variables used means and standard deviations. Using chi-square or Fisher exact test, we conducted the univariate analysis to identify the AMS strategies implemented and the challenges associated with adopting ASP between hospitals with ASP and those without ASP. P-values less than 0.05 were considered statistically significant. Estimates of ASP in hospitals were presented using percentages and their corresponding 95% confidence intervals.

RESULTS

Characteristics of respondents

A total of 32 out of 43 hospitals participated, generating a response rate of 74.4%. Most heads of IPCs who responded were pharmacists (17/32,53.1%), followed by medical specialists (6/32,18.8%), medical officers (5/32, 15.6%), and pharmacy technicians (4/32, 12.5%). The mean age and standard deviation (SD) of IPC heads who responded were 36.1 (±1.1) years. Most heads of IPC in hospitals that participated had a bachelor's degree as their highest level of education. District hospitals had the

highest proportion of participation (66%), followed by regional referral (25%) and, lastly, PNFPs (9%). Eleven out of 43 (25.6%) hospitals declined to participate in this study. Seven of the eleven health facilities were located in the north, two in the central region, one in the Eastern, and one in the Western (**Table 1).**

Table 1. Characteristics of the study population (N=32).

**Description**	**Frequency**	**Percentage**
	**(N = 32)**	**100%**
**Type of cadre**		
Pharmacists	17	53.1
Medical officers	5	15.6
Medical specialist	6	18.8
Pharmacy Technicians	4	12.5
**Type of health facility**		
Regional referral hospitals	8	25
District hospitals	21	65.6
Private not for profit	3	9.4
**Teaching status of the hospital**		
Teaching hospitals	11	34.4
Non-teaching hospitals	21	65.6

**Region**		
Central	10	31.3
North	4	12.5
East	9	28.1
West	9	28.1
**hospital bed capacity**		
100 beds	21	65.6
101- 300	3	9.4
Over 300	8	25

Characteristics of antimicrobial stewardship programmes in hospitals

ASPs were implemented in 14 of the 32 (44 %, 95% CI 26-62) studied hospitals. Five of the fourteen (36%) hospitals with ASP were located in the eastern region, followed by western 29% (4/14), central with 21% (3/14), and northern with 14% (2/14). Half of the hospitals (7/14, 50%) with ASP were regional referral hospitals. Over 63% (9/14) of hospitals with ASP were teaching hospitals. Eight out of the 14 (57%) hospitals with ASP had a bed capacity of 300 beds and above (Table 2).

Table 2. Characteristics of hospitals with or without an Antimicrobial stewardship programme (N = 32).

	Hospital with ASP	Hospitals without ASP	Total

	**n = 14**	**n=18**	**N=32**
	**n(%)**	**n(%)**	**n(%)**
**Region**			
Central	3 (21.4)	7 (38.9)	10 (31.3)
North	2 (14.3)	2 (11.1)	4 (12.5)
East	5 (35.7)	4 (22.2)	9 (28.1)
West	4 (28.6)	5 (27.8)	9 (28.1)
**Type of health facility**			
Regional referral hospital	7 (50)	1 (5.6)	8 (25)
District hospital	5 (35.7)	16 (88.9)	21 (65.6)
Private-not-for-profit	2 (14.3)	1 (5.6)	3 (9.4)
**Nature of the health facility**			
Teaching hospital	9(64.3)	2 (11.1)	11 (34.4)
Non-teaching hospital	5(35.7)	16 (88.9)	21 (65.6)
**Bed capacity**			
100 beds	5 (35.7)	14 (77.8)	21 (65.6)
101- 300	1 (7.1)	2 (11.1)	3 (9.4)
Over 300	8 (57.1)	2 (11.1)	8 (25)

Abbreviations, ASP: Antimicrobial Stewardship programmes, %: per cent

Antimicrobial stewardship strategies reported in hospitals

Documentation of antibacterial use in medical charts was the most implemented (30/32, 93.3%) among the two AMS policy strategies. All 14 (100%) hospitals with ASP documented antibacterials use, as compared to 11 out of 18 (89 %) without ASP, though not significantly different (P=0.198).

Pre-authorisation and approval were the most frequently implemented (22/32, 68.8%) of the three core AMS strategies, with 11 out of 14 (78.6%) hospitals with ASP implementing the AMS strategy, compared to 11 out of 18 (61.1 %) hospitals without ASP (P=0.290).

In all 32 hospitals, antimicrobial combination therapy was the most commonly used (31/32, 96.9 %). Antimicrobial combination therapy was implemented in all 14 (100%) hospitals with ASP, compared to 17 out of 18 (94.4%) hospitals without ASP (P= 0.370).

There was a statistically significant difference between hospitals with and without ASP in terms of implementing AMS strategies such as antibacterial time-out AMS policy strategy (P=0.014) and supplementary AMS strategies such as streamlining or de-escalation (P=0.025), reporting of culture and sensitivity results (P=0.016), and hospital-developed clinical decision-support systems (P=0.043).

Antibiograms were the least performed supplementary AMS strategy performed in 9% (3/32) hospitals. Only two out of 14 (14.3%) hospitals with ASP developed antibiograms, while one out of 18 (5.6%) hospitals without ASP developed antibiograms (**Table 3**).

Table 3. Antimicrobial stewardship strategies reported in hospitals with or without ASP in Uganda (N = 32).

	**Hospitals with ASP**	**Hospitals without ASP**	**Total**	**P-value**

	(n =14)	n=18	N =32	
	n(%)	n(%)	N (100 %)	
**1) AMS Policy strategies**				
Documentation of antibacterials use in medical charts	14 (100)	16 (88.9)	30 (93.8)	0.198
Antibacterial time-out after 48 to 72 hour	10 (71.4)	5 (27.8)	15 (46.9)	0.014*
**2) AMS Core strategies**				
Pre-authorization and approval technique	11 (78.6)	11 (61.1)	22 (68.8)	0.290
Prospective audit with Feedback	9 (64.3)	8 (44.4)	17 (53.1)	0.265
Antimicrobial formulary restriction and approval systems	7 (50)	5 (27.8)	12 (37.5)	0.198
**3) Supplemental AMS strategies**				
Antimicrobial order forms	7 (50)	4 (22.2)	11 (34.4)	0.101
Antimicrobial combination therapy	14 (100)	17 (94.4)	31 (96.9)	0.370
Streamlining or de-escalation (discontinuing treatment if no bacterial infection	11 (78.6)	7 (38.9)	18 (56.3)	0.025*
Dose optimisation of antibacterial	13 (92.9)	12 (66.7)	25 (78.1)	0.075

A systematic plan for conversion of parenteral to oral (Intravenous (IV) to oral (PO))	13 (92.9)	14 (77.8)	27 (84.4)	0.244
Standard treatment guidelines and clinical pathways	13 (92.9)	16(88.9)	29 (90.6)	0.702
Use of education strategies to educate prescribers on appropriate prescribing	13 (92.9)	12 (66.7)	25 (78.1)	0.075
Diagnostic pathways for patients with reported bacterial infection	9 (64.3)	12 (66.7)	21 (65.6)	0.888
Developing antibiograms	2 (14.3)	1 (5.6)	3 (9.4)	0.401
Use rapid diagnostic tests	9 (64.3)	10 (55.6)	19 (59.4)	0.618
Hospital monitor antimicrobial resistance	7 (50)	5 (27.8)	12 (37.5)	0.198
Reporting of culture and sensitivity results	9 (64.3)	4 (22.2)	13 (40.6)	0.016*
Education on good antimicrobial prescribing practice and resistance	12 (85.7)	14 (77.8)	26 (81.3)	0.568
Hospital developed Clinical decision-support systems	8 (57.1)	4 (22.2)	12 (37.5	0.043*

AMS: Antimicrobial stewardship, ASP: Antimicrobial stewardship programme, IV: Intravenous, PO: oral

Challenges in the implementation of an ASP in selected hospitals in Uganda

In this study, the most reported ASP adoption or implementation challenges included a lack of time 91% (29/32) and dedicated funds for the ASP team 91% (29/32). However, a lack of adequate training and education was most reported by hospitals with ASP, while for those hospitals without ASP, the most frequently reported challenge was a lack of time among the AMS team and a lack of dedicated funding for the AMS team (**Table 4**).

Table 4. Challenges of implementing ASP in hospitals with or without an ASP (N = 32).

	**Hospitals with ASP**	**Hospitals without ASP**	**Total**	**P-value**
	**(n =14)**	**n=18**	**N = 32**	
	**n(%)**	**n(%)**	**n(%)**	
Lack of training and education in antimicrobial stewardship	13 (92.9)	14 (77.8)	27 (84.4)	0.244
Lack of time amongst antimicrobial stewardship programme team	12(85.7)	17 (94.4)	29 (90.6	0.401

Lack of dedicated funding for an antimicrobial stewardship team	12(85.7)	17 (94.4)	29 (90.6	0.401
Lack of leadership to promote antimicrobial stewardship at the facility	10 (71.4)	11 (61.1)	21 (65.6)	0.542
Lack of support from experienced senior clinicians at the facility	6 (42.9)	10 (55.6)	16 (50)	0.476
Lack of infectious diseases or microbiology services	7 (50)	12 (66.7)	19 (59.4)	0.341
Lack of pharmacy resources	5(35.7)	9 (50)	14 (43.8)	0.419
Lack of willingness from healthcare providers to change their prescribing practices	8 (57.1)	10 (55.6)	18 (56.3)	0.928

Lack of enforcement by facility management/executive	7 (50)	13 (72.2)	20 (62.5)	0.198
Lack of an electronic medication management system	9 (64.3)	12 (66.7)	21 (65.6)	0.888
High level of transient or part-time staff	5 (35.7)	8(44.4)	13 (40.6)	0.618
Inadequate time for Antimicrobial stewardship activities	11 (78.6)	13 (72.2)	24 (75)	0.681
Personnel and expertise shortages	7 (50)	14 (77.8)	21 (65.6)	0.101
Inadequate funding [illegible]r AMS activities	11(78.6)	13 (72.2)	24 (75)	0.681
Lower priority for AMS than other clinical initiatives	11 (78.6)	15 (83.3)	26 (81.3)	0.732
Inadequate information technology (IT) support for antimicrobial stewardship	11 (78.6)	13 (72.2)	24 (75)	0.681

Opposition of AMS by healthcare providers	8 (57.1)	8 (44.4)	16 (50)	0.476
A paucity of data on improved outcomes with ASPs in Uganda	11 (78.6)	15 (83.3)	26 (81.3)	0.732
Lack of policy support for ASP activities from government or other partners	12 (85.7)	15 (83.3)	27 (84.4)	0.854

Abbreviations, ASP: Antimicrobial Stewardship programmes, %: percent

DISCUSSION

The Uganda national action plan (NAP) on AMR (2018-2023) is under implementation, emphasising optimising antibacterial use through strengthening and operationalising ASP among all hospitals regardless of their size[32]. While many healthcare providers from public and non-profit hospitals have received AMS training to help them operationalise and strengthen ASPs in their facilities, there is limited information on the characteristics of ASP, the AMS strategies used, and the challenges associated with ASP implementation[28].

In this survey, only 44 % of the hospitals implemented ASPs in their hospitals. Our study found a higher prevalence of adopted ASPs than a recent study in Nigeria, which found that 35% of the 17

hospitals in six different Nigerian regions implemented ASPs [33]. The difference in the proportion of ASP in our study can be explained by the number of participating hospitals (32 versus 17) and their difference in types and teaching status[33]. Furthermore, our findings show that ASPs were distributed in tertiary hospitals (regional referral hospitals) and district hospitals and PNFPs, contrary to the Nigerian study that only reported ASP in Tertiary hospitals. Previous studies indicated that larger hospitals (tertiary or regional referrals) were more likely to implement ASP than smaller hospitals due to the availability of more resources in the former than the latter, which contrasts our findings [1, 34]. The distribution of ASP in different types of hospitals (regional referrals, district hospitals, and PNFPs) of Uganda could be due to the Ministry of Health and other development partners continued strengthening and operationalisation of ASP and medicines and therapeutics committees in public hospitals and PNFPs. The low adoption of ASPs without any formal guidelines indicates that hospitals have recognised their beneficial effects in optimising antibacterial use, and some of the trained healthcare providers are spearheading their introduction from the available international guidelines of CDC and WHO and local capacity development from one health initiative [12, 20, 28]. This finding will go a long way in supporting the dissemination strategy of the national action plan on AMR and supporting national guidelines on developing ASP [32].

Regardless of the absence or presence of an ASP, hospitals in our study implemented AMS strategies broadly. At least one AMS strategy was conducted in over 96% of hospitals surveyed. This finding was consistent with previous studies conducted in the United States that reported that over 90% of hospitals implemented at least one AMS strategy despite having an ASP or not [11, 35, 36]. However, it contrasts with previous Nigerian studies, where only 72% of hospitals performed at least one AMS strategy [37, 38]. The majority of the hospitals implemented documenting antibacterial in the medical

charts, pre-authorisation and approval of antibacterial and antimicrobial combination therapy as dominant policy, core and supplementary AMS strategies respectively, where the difference of implementing these AMS strategies between hospitals with or without ASP was not statistically significant. It has been shown in previous studies that it is exactly hard to predict the many AMS strategies implemented which could be most effective in optimising antibacterial use [36, 39]. However, the number of strategies used by an ASP may be more predictive of reductions in targeted antibacterial use than the total amount of resources available to the ASP[35].

In our study, pre-authorisation and approval were the most frequently reported AMS core strategy (68%) among the 32 hospitals. This finding is similar to a previous study in Nigeria, where 12% of 17 tertiary hospitals conducted pre-authorisation as a common core AMS strategy [33]. The drivers influencing hospitals with or without ASP adopting pre-authorisation in our study may not be clear. However, other studies in Africa have alluded to having limited resources, thus using the core strategy to reserve certain antibacterials among all healthcare providers [33]. However, our findings may contrast the Infectious Diseases Society of America (IDSA) recommendations and findings of previous studies that reported post prospective audit and feedback as the most performed AMS strategy. The strategy was less cumbersome to prescribers, and it does not create a barrier to initiation of therapy and is more acceptable to prescribers [7, 40]. This finding might imply that policymakers' adoption of core AMS strategy has to consider the availability of a certain staffing level, resources, and region before recommending specific AMS strategies to hospitals.

A lack of dedicated funding and time for the ASP team were the most frequently reported challenges to adopt an ASP in hospitals without ASP and those implementing ASP. A report by the World Health

Organization (WHO) on the ability of LMICs to implement the AMS tool kit highlighted similar issues as a barrier to AMS implementation to include a lack of human and financial resources and insufficient training [41]. In addition, other reviews have identified a lack of diagnostic facilities, poor laboratory service infrastructure, a high incidence of infectious illnesses, and a lack of suitable staffing levels as significant barriers to ASP implementation in Uganda [41, 42]. Given this finding, the national steering committee on AMS must consider these issues while developing guidelines and frameworks for ASP implementation in Uganda.

limitations

Our research was cross-sectional, with respondents from various types of hospitals in various regions. We could not verify the accuracy or completeness of responses by utilising at least one head or chair of the infection control committee. However, the position of head of the infection control committee and their active involvement in hospital activities may have regulated their overestimation or underestimation of responses about the ASP. Even after differentiating between hospitals with and without ASP coverage, there was no statistically significant difference in adopting core AMS strategies, suggesting that the distinction between hospitals with and without ASP coverage may have been blurred. Our statistical study was descriptive in determining the characteristics of ASP and corresponding AMS strategies in those hospitals with or without ASP. Due to the small sample size, we could not identify factors associated with ASP adoption in Uganda. Despite these limitations, the present study offers insight into hospitals' progress in adopting ASP and AMS strategies regardless of the availability of guidelines on ASP. It further confirms that there has been some benefit in the ongoing capacity building programmes to operationalise ASP in Uganda. The findings highlight the need for the Ugandan government to support ASP in all Ugandan hospitals.

Conclusions

The majority of hospitals in Uganda have implemented at least one AMS strategy to optimise antibacterial use despite the low coverage of ASP. Pre-authorization was the most adopted AMS core strategy in hospitals despite their ASP status. The ministry of health needs to sensitise and support the establishment of ASP in hospitals across the countries.

Supporting information

S1_Checklist. STROBE statement Checklist of items included in cross-sectional studies.

(DOC)

S2_Appendix. Informed consent form.

(DOCX)

S3_ Appendix. Questionnaire

(DOCX)

S4_Map of the study setting.

(pdf)

S5_Appendix. Approval letters of ethical clearance.

(DOCX)

List of abbreviations

AMS, Antimicrobial Stewardship; ASP, Antimicrobial Stewardship Programmes; WHO, World Health Organization; GOU, Government of Uganda; MOH, Ministry of Health

Acknowledgements

The authors want to thank Prof Jasper Ogwal Okeng and Dr Jackson Mukonzo for their support in this work. We want to thank the 32 heads of the infection control committee from the eight regional referrals hospitals, 21 general hospitals, and three private-not for-profit facilities. Finally, we are grateful to all the research assistants who supported us in the data collection in this study.

Ethics approval and consent to participate

The Makerere University School of Biomedical Sciences Higher Degree Research and Ethics Committee approved our study procedure (SBH-HDREC-624). The Uganda National Council of Science and Technology (UNCST) granted further ethical approval for the project (HS339ES). All respondents involved in this study provided written informed consent. A consent form was included in the questionnaire, outlining the study goal and ensuring respondents' privacy and confidentiality, as well as the voluntary nature of their participation.

Consent for publication

Not applicable

Availability of data and materials

The study's datasets used in the analysis are available from the corresponding author on request.

Competing interests

Not applicable

Funding

This study obtained funding through the Makerere University-Swedish International Development Agency (SIDA) collaboration (Sida PI0010). However, the funders never participated in the study design, data collection and analysis, publishing decisions, or manuscript preparation.

Authors' contribution

The authors participated in this submitted manuscript's joint development, performing critical roles from early conceptualisation to producing the entire manuscript. The manuscript resulted from a collaborative effort where all authors read and approved the final manuscript

Conceptualisation and design: Isaac Kimbowa, Joan N. Kalyango, Moses Ocan, Celestino Obua, Cecilia Stålsby Lundborg, Jaran Eriksen),

Data curation and analysis: Isaac Kimbowa, Joan N. Kalyango, Moses Ocan. Jaran Eriksen,

Funding acquisition: Isaac Kimbowa, Cecilia Stålsby Lundborg, Celestino Obua, Cecilia Stålsby Lundborg

Investigation: Isaac Kimbowa, Moses Ocan, Joan N. Kalyango, Cecilia Stålsby Lundborg.

Methodology: Isaac Kimbowa, Joan N. Kalyango, Moses Ocan, Cecilia Stålsby Lundborg, Jaran Eriksen, Celestino Obua,

Supervision: Joan N. Kalyango, Moses Ocan, Jaran Eriksen, Celestino Obua, Cecilia Stålsby Lundborg. **Writing, review and editing**: Isaac Kimbowa, Moses Ocan, Joan N. Kalyango, Cecilia Stålsby Lundborg Jaran Eriksen, Celestino Obua.

References

1. Cantey JB, Vora N, Sunkara M. Prevalence, Characteristics, and Perception of Nursery Antibiotic Stewardship Coverage in the United States. J Pediatric Infect Dis Soc. 2017;6(3):e30-e5. doi: https://doi.org/10.1093/jpids/piw040. PubMed PMID: 27422868; PubMed Central PMCID: PMCPMID: 27422868.
2. Pollack LA, van Santen KL, Weiner LM, Dudeck MA, Edwards JR, Srinivasan A. Antibiotic Stewardship Programs in U.S. Acute Care Hospitals: Findings From the 2014 National Healthcare Safety Network Annual Hospital Survey. Clin Infect Dis. 2016;63(4):443-9. DOI: https://doi.org/10.1093/cid/ciw323. PubMed PMID: 27199462; PubMed Central PMCID: PMCPMID: 27199462 PMCID: PMC6537894.
3. Pollack LA, Srinivasan A. Core elements of hospital antibiotic stewardship programs from the Centers for Disease Control and Prevention. Clinical Infectious Diseases. 2014;59(suppl_3):S97-S100. DOI: https://dx.doi.org/10.1093%2Fcid%2Fciu542. PubMed Central PMCID: PMCPMCID: PMC6521960 PMID: 25261548.
4. Kubin CJ. Antimicrobial Stewardship Programs. Disease Management & Health Outcomes. 2008;16(6):403-10. DOI: https://doi.org/10.1093/ajhp/zxaa419. PubMed Central PMCID: PMCPMID: 33543233 PMCID: PMC7929392.
5. Stenehjem E, Hyun DY, Septimus E, Yu KC, Meyer M, Raj D, et al. Antibiotic stewardship in small hospitals: barriers and potential solutions. Clinical Infectious Diseases. 2017;65(4):691-6.
6. Charani E, Holmes A. Antibiotic stewardship—twenty years in the making. Antibiotics. 2019;8(1):7. DOI: https://doi.org/10.3390/antibiotics8010007. PubMed Central PMCID: PMCPMID: 30678365 PMCID: PMC6466570.
7. Shlaes DM, Gerding DN, John Jr JF, Craig WA, Bornstein DL, Duncan RA, et al. Society for Healthcare Epidemiology of America and Infectious Diseases Society of America Joint Committee on

the Prevention of Antimicrobial Resistance: guidelines for the prevention of antimicrobial resistance in hospitals. Clinical infectious diseases. 1997;25(3):584-99. DOI: https://doi.org/10.1086/513766.

8. Dyar O, Huttner B, Schouten J, Pulcini C. What is antimicrobial stewardship? Clinical microbiology and infection. 2017;23(11):793-8. DOI: https://doi.org/10.1016/j.cmi.2017.08.026. PubMed Central PMCID: PMCPMID: 28882725.

9. Howard P, Pulcini C, Levy Hara G, West RM, Gould IM, Harbarth S, et al. An international cross-sectional survey of antimicrobial stewardship programmes in hospitals. J Antimicrob Chemother. 2015;70(4):1245-55. doi: https://doi.org/10.1093/jac/dku497. PubMed PMID: 25527272; PubMed Central PMCID: PMCPMID: 25527272.

10. Dellit TH, Owens RC, McGowan JE, Gerding DN, Weinstein RA, Burke JP, et al. Infectious Diseases Society of America and the Society for Healthcare Epidemiology of America guidelines for developing an institutional program to enhance antimicrobial stewardship. Clinical infectious diseases. 2007;44(2):159-77. doi: https://doi.org/10.1086/510393. PubMed Central PMCID: PMCPMID: 17173212.

11. Doron S, Nadkarni L, Lyn Price L, Kenneth Lawrence P, Davidson LE, Evans J, et al. A nationwide survey of antimicrobial stewardship practices. Clin Ther. 2013;35(6):758-65 e20. DOI: https://doi.org/10.1016/j.clinthera.2013.05.013. PubMed PMID: 23795573; PubMed Central PMCID: PMCPMID: 23795573.

12. CDC. The Core Elements of Human Antibiotic Stewardship Programs in Resource-Limited Settings: National and Hospital Levels. In: US Department of Health and Human Services C, editor. Atlanta, GA: CDC; 2018.

13. Anderson DJ, Watson S, Moehring RW, Komarow L, Finnemeyer M, Arias RM, et al. Feasibility of core antimicrobial stewardship interventions in community hospitals. JAMA network open. 2019;2(8):e199369-e. Doi: https://doi.org/10.1001/jamanetworkopen.2019.9369. PubMed Central PMCID: PMCPMID: 31418804 PMCID: PMC6704742.

14. Fishman N, America SfHEo, America IDSo. Policy statement on antimicrobial stewardship by the society for healthcare epidemiology of America (SHEA), the infectious diseases society of America (IDSA), and the pediatric infectious diseases society (PIDS). Infection Control & Hospital

Epidemiology. 2012;33(4):322-7. DOI: https://doi.org/10.1086/665010. PubMed Central PMCID: PMCPMID: 22418625.

15. Dyar O, Pagani L, Pulcini C. Strategies and challenges of antimicrobial stewardship in long-term care facilities. Clinical Microbiology and Infection. 2015;21(1):10-9. DOI: https://doi.org/10.1016/j.cmi.2014.09.005. PubMed Central PMCID: PMCPMID: 25636921.

16. Pierce J, Apisarnthanarak A, Schellack N, Cornistein W, Al Maani A, Adnan S, et al. Global antimicrobial stewardship with a focus on low-and middle-income countries. International Journal of Infectious Diseases. 2020. DOI: https://doi.org/10.1016/j.ijid.2020.05.126. PubMed Central PMCID: PMCPMCID: PMC7271868 PMID: 32505875.

17. Ackers L, Ackers-Johnson G, Seekles M, Odur J, Opio S. Opportunities and challenges for improving antimicrobial stewardship in low-and middle-income countries; lessons learnt from the maternal sepsis intervention in Western Uganda. Antibiotics. 2020;9(6):315. DOI: https://dx.doi.org/10.3390%2Fantibiotics9060315. PubMed Central PMCID: PMCPMC: 7345100 PMID: 32526969.

18. Majumder MAA, Rahman S, Cohall D, Bharatha A, Singh K, Haque M, et al. Antimicrobial Stewardship: Fighting Antimicrobial Resistance and Protecting Global Public Health. Infection and drug resistance. 2020;13:4713. DOI: https://dx.doi.org/10.2147%2FIDR.S290835. PubMed Central PMCID: PMCPMCID: PMC7778387 PMID: 33402841.

19. World-Health-Organisation. Global action plan on antimicrobial resistance. World Health Organization Geneva, Switzerland; 2015.

20. World-Health-Organisation. Antimicrobial stewardship programmes in health-care facilities in low-and middle-income countries: a WHO practical toolkit. 2019.

21. Munkholm L, Rubin O. The global governance of antimicrobial resistance: a cross-country study of alignment between the global action plan and national action plans. Globalisation and health. 2020;16(1):1-11. DOI: https://doi.org/10.1186/s12992-020-00639-3.

22. Akinboyo IC, Gerber JS, editors. Principles, Policy and Practice of Antibiotic Stewardship. Seminars in Perinatology; 2020: Elsevier.

23. da Silva AA, de Almeida Dias DA, Marques A, di Biase CB, Murni IK, Dramowski A, et al. Role of antimicrobial stewardship programmes in children: a systematic review. Journal of Hospital Infection. 2018;99(2):117-23.
24. Ibrahim NH, Maruan K, Khairy HAM, Hong YH, Dali AF, Neoh CF. Economic evaluations on antimicrobial stewardship programme: a systematic review. Journal of Pharmacy & Pharmaceutical Sciences. 2017;20:397-406. DOI: https://doi.org/10.18433/j3nw7g. PubMed Central PMCID: PMCPMID: 29145934.
25. Kapadia SN, Abramson EL, Carter EJ, Loo AS, Kaushal R, Calfee DP, et al. The expanding role of antimicrobial stewardship programs in hospitals in the United States: lessons learned from a multisite qualitative study. The Joint Commission Journal on Quality and Patient Safety. 2018;44(2):68-74. DOI: https://doi.org/10.1016/j.jcjq.2017.07.007.
26. Mas-Morey P, Valle M. A systematic review of inpatient antimicrobial stewardship programmes involving clinical pharmacists in small-to-medium-sized hospitals. European Journal of Hospital Pharmacy. 2018;25(e1):e69-e73. DOI: http://orcid.org/0000-0002-3515-251X.
27. Huebner C, Flessa S, Huebner N. The economic impact of antimicrobial stewardship programmes in hospitals: a systematic literature review. Journal of Hospital Infection. 2019;102(4):369-76. DOI: https://doi.org/10.1016/j.jhin.2019.03.002. PubMed Central PMCID: PMCPMID: 30880265.
28. Musoke D, Kitutu FE, Mugisha L, Amir S, Brandish C, Ikhile D, et al. A One Health Approach to Strengthening Antimicrobial Stewardship in Wakiso District, Uganda. Antibiotics. 2020;9(11):764. doi: https://doi.org/10.3390/antibiotics9110764.
29. Ministry-of-Health. Medicines and Therapeutics committee manual. 2018:169.
30. Ministry-of-Health. National Health Facility Master List 2018. A complete list of all health facilities in Uganda. Kampala , Uganda 2018.
31. Mpaata KA, Bumali L, Okiria JC. Availability of Specialists in Hospitals and the Health Care Delivery System in Uganda. 2017.
32. Goverment-of-Uganda. Uganda National Action Plan for Antimicrobial Resistance 2018-2023. In: Minister of Health MoA, Animal Industry, and Fisheries and Minster of Water and the Environment, editor. 2018. p. 115.

33. Fadare JO, Ogunleye O, Iliyasu G, Adeoti A, Schellack N, Engler D, et al. Status of antimicrobial stewardship programmes in Nigerian tertiary healthcare facilities: findings and implications. Journal of global antimicrobial resistance. 2019;17:132-6. doi: https://doi.org/10.1016/j.jgar.2018.11.025. PubMed Central PMCID: PMCPMID: 30557686.

34. van Limburg M, Sinha B, Lo-Ten-Foe JR, van Gemert-Pijnen JE. Evaluation of early implementations of antibiotic stewardship program initiatives in nine Dutch hospitals. Antimicrobial resistance and infection control. 2014;3(1):33. DOI: https://doi.org/10.1186/2047-2994-3-33. PubMed Central PMCID: PMCPMID: 25392736 PMCID: PMC4228167.

35. Chou AF, Graber CJ, Jones M, Zhang Y, Goetz MB, Madaras-Kelly K, et al. Characteristics of antimicrobial stewardship programs at Veterans Affairs hospitals: results of a nationwide survey. Infection control and hospital epidemiology. 2016;2016:1-8. DOI: https://doi.org/10.1017/ice.2016.26. PubMed Central PMCID: PMCPMID: 26905338.

36. Fu CJ, Mantell E, Stone PW, Agarwal M. Characteristics of nursing homes with comprehensive antibiotic stewardship programs: Results of a national survey. American Journal of Infection Control. 2020;48(1):13-8. DOI: https://doi.org/10.1016/j.ajic.2019.07.015. PubMed Central PMCID: PMCPMID: 31447117 PMCID: PMC6935405.

37. Chukwu EE, Oshun PO, Osuolale KA, Chuka-Ebene VO, Salako A, Idigbe IE, et al. Antimicrobial stewardship programmes in healthcare facilities in Lagos State, Nigeria: a needs assessment. Journal of global antimicrobial resistance. 2021;25:162-70. DOI: https://doi.org/10.1016/j.jgar.2021.02.034. PubMed Central PMCID: PMCPMID: 33812050.

38. Abubakar U, Tangiisuran B. Nationwide survey of Pharmacist’s involvement in antimicrobial stewardship program in Nigerian Tertiary Hospitals. Journal of Global Antimicrobial Resistance. 2019. doi: https://doi.org/10.1016/j.jgar.2019.10.007. PubMed Central PMCID: PMCPMID: 31628999.

39. Doron S, Nadkarni L, Price LL, Lawrence PK, Davidson LE, Evans J, et al. A nationwide survey of antimicrobial stewardship practices. Clinical therapeutics. 2013;35(6):758-65. e20. doi: https://doi.org/10.1016/j.clinthera.2013.05.013.

40. Jacobs DM, Kuper K, Septimus E, Arafat R, Garey KW. Assessment of antimicrobial stewardship activities in a large metropolitan area. Journal of pharmacy practice. 2016;29(3):188-93. doi: https://doi.org/10.1177/0897190014549842. PubMed Central PMCID: PMCPMID: 25312262.

41. Maki G, Smith I, Paulin S, Kaljee L, Kasambara W, Mlotha J, et al. Feasibility Study of the World Health Organization Health Care Facility-Based Antimicrobial Stewardship Toolkit for Low- and Middle-Income Countries. Antibiotics. 2020;9(9):556.

42. Cox JA, Vlieghe E, Mendelson M, Wertheim H, Ndegwa L, Villegas MV, et al. Antibiotic stewardship in low-and middle-income countries: the same but different? Clinical microbiology and infection. 2017;23(11):812-8. doi: :https://doi.org/10.1016/j.cmi.2017.07.010. PubMed Central PMCID: PMC28712667.

Figure

Click here to access/download
Supporting Information
S4_Map of the study setting .pdf

Click here to access/download
Supporting Information
S1_ Appendix. STROBE Statement ..docx

Click here to access/download
Supporting Information
S3_Questionaire for this manuscript.docx

Click here to access/download
Supporting Information
S6_Appendix.UNCST ethical clearance Letter.pdf

Click here to access/download

Supporting Information

S5_Appendix. Approval letters Makerere University .docx

Click here to access/download
Supporting Information
S2_Appendix. Informed consent .docx
