# Supplementary material for: Characteristics of antimicrobial stewardship programmes in hospitals of Uganda
Source: PLoS One. 2022 May 10;17(5):e0268032. doi: 10.1371/journal.pone.0268032 (PMC9089898; doi:10.1371/journal.pone.0268032)
Supplement: Attachment — Submitted filename: Response to reviewer comments PONE.docx [file pone.0268032.s005.docx]

**RESPONSE TO REVIEWER'S COMMENTS ON MANUSCRIPT, PONE-D-21-34980**

Dear Editor:

We appreciate the comments. We have revised our manuscript according to the editor and reviewers' comments, questions, and suggestions. We appreciate the comments and feedback since they have improved our manuscript. We are grateful for your service.

**Yours,**

**Isaac Kimbowa**

**Corresponding author**

**RESPONSE TO REVIEWER'S COMMENTS ON MANUSCRIPT, PONE-D-21-34980**

**Comment # 01 Editor:** When submitting your revision, we need you to address these additional requirements. 1. Please ensure that your manuscript meets PLOS ONE's style requirements, including those for file naming. The PLOS ONE style templates can be found at

<https://journals.plos.org/plosone/s/file?id=wjVg/PLOSOne_formatting_sample_main_body.pdf> and <https://journals.plos.org/plosone/s/file?id=ba62/PLOSOne_formatting_sample_title_authors_affiliations.pdf>

**Response**

**We have abided with all PLOS ONE’s styles requirement including filing requirements**

**Comment # 02, Editor :** 2. We note that the grant information you provided in the ‘Funding Information’ and ‘Financial Disclosure’ sections do not match. When you resubmit, please ensure that you provide the correct grant numbers for the awards you received for your study in the ‘Funding Information’ section.

**Response**

**The above grant number provided is the correct one because the study received funding through the Makerere University-Swedish International Development Agency (SIDA) collaboration (Sida PI0010).**

**Comment # 03, Editor :** 3. Thank you for stating the following financial disclosure: “No-The funders had no role in study design, data collection and analysis, decision to publish, or preparation of the manuscript.” Please state what role the funders took in the study.  If the funders had no role, please state: "The funders had no role in study design, data collection and analysis, decision to publish, or preparation of the manuscript." statement is not correct you must amend it as needed.

**Response**

**We have amended the funder’s statement**

Please include this amended Role of Funder statement in your cover letter; we will change the online submission form on your behalf.

**Response**

**We have included the amended role of funder statement in our cover letter**

**Comment # 04, Editor :** 4. Thank you for stating the following in the Acknowledgments Section of your manuscript:

“This study obtained funding through the Makerere University-Swedish International Development Agency (SIDA) collaboration (Sida PI0010). However, the funders never participated in the study design, data collection and analysis, publishing decisions, or manuscript preparation.”

**Response**

**We have amended the funding information….. lines 382, page 26**

We note that you have provided additional information within the Acknowledgements Section that is not currently declared in your Funding Statement. Please note that funding information should not appear in the Acknowledgments section or other areas of your manuscript. We will only publish funding information present in the Funding Statement section of the online submission form.

**Response**

**We have removed all funding text in acknowledgement section …. lines 364-368,pages 25**

Please remove any funding-related text from the manuscript and let us know how you would like to update your Funding Statement. Currently, your Funding Statement reads as follows: “No-The funders had no role in study design, data collection and analysis, decision to publish, or preparation of the manuscript.”

**Response**

**We have amended the funding statement and we would like it to be captured as written in the manuscript**

Please include your amended statements within your cover letter; we will change the online submission form on your behalf.

**Response**

**We have included the amended statement in our cover letter**

**Comment # 05, Editor :** 5. We note that you have stated that you will provide repository information for your data at acceptance. Should your manuscript be accepted for publication, we will hold it until you provide the relevant accession numbers or DOIs necessary to access your data. If you wish to make changes to your Data Availability statement, please describe these changes in your cover letter and we will update your Data Availability statement to reflect the information you provide.

**Response**

**We don’t have any changes in the data availability statement. We like to maintain our data availability statement**

**Comment # 06, Editor :**6. Your ethics statement should only appear in the Methods section of your manuscript. If your ethics statement is written in any section besides the Methods, please move it to the Methods section and delete it from any other section. Please ensure that your ethics statement is included in your manuscript, as the ethics statement entered into the online submission form will not be published alongside your manuscript.

**Response**

**We have only maintained the ethics statements in the manuscript only**

**Comment # 07, Editor :**7. Please include captions for your Supporting Information files at the end of your manuscript, and update any in-text citations to match accordingly. Please see our Supporting Information guidelines for more information: <http://journals.plos.org/plosone/s/supporting-information>.

**Response**

**We have included captions for our supporting information files and updated our in-text citations in the manuscript**

[Note: HTML markup is below. Please do not edit.]

Reviewers' comments:

Reviewer's Responses to Questions

**Comments to the Author**

**Comment # 01, Reviewers**

1. Is the manuscript technically sound, and do the data support the conclusions?

The manuscript must describe a technically sound piece of scientific research with data that supports the conclusions. Experiments must have been conducted rigorously, with appropriate controls, replication, and sample sizes. The conclusions must be drawn appropriately based on the data presented.

Reviewer #1: Partly

Reviewer #2: Yes

**Comment # 02, Reviewers**

2. Has the statistical analysis been performed appropriately and rigorously?

Reviewer #1: Yes

Reviewer #2: Yes

**Comment # 03, Reviewers**

3. Have the authors made all data underlying the findings in their manuscript fully available?

The [PLOS Data policy](http://www.plosone.org/static/policies.action#sharing) requires authors to make all data underlying the findings described in their manuscript fully available without restriction, with rare exception (please refer to the Data Availability Statement in the manuscript PDF file). The data should be provided as part of the manuscript or its supporting information, or deposited to a public repository. For example, in addition to summary statistics, the data points behind means, medians and variance measures should be available. If there are restrictions on publicly sharing data—e.g. participant privacy or use of data from a third party—those must be specified.

Reviewer #1: Yes

Reviewer #2: Yes

**Comment # 04, Reviewers**

4. Is the manuscript presented in an intelligible fashion and written in standard English?

PLOS ONE does not copyedit accepted manuscripts, so the language in submitted articles must be clear, correct, and unambiguous. Any typographical or grammatical errors should be corrected at revision, so please note any specific errors here.

Reviewer #1: Yes

Reviewer #2: No

5. Review Comments to the Author

Please use the space provided to explain your answers to the questions above. You may also include additional comments for the author, including concerns about dual publication, research ethics, or publication ethics. (Please upload your review as an attachment if it exceeds 20,000 characters)

**Response**

**noted**

**Reviewer 1**

**Comment # , Reviewer 1:** Reviewer #1: Thank you for the opportunity to review your manuscript. Please see below a few comments to further enhance your manuscript.

**Comment # 01, Reviewer 1:** 1.Abstract and introduction.•Please ensure all acronyms and abbreviations are defined at first mentioned. You also need to be consistent in the use of these acronyms/abbreviations.

**Response**

**We have corrected all the abbreviations and defined acronyms , lines 2 ,page 2;**

**Comment # 02, Reviewer 1:** 2.Methodology. •There is a discrepancy in the survey timeline. In the abstract you stated that the survey was conducted b/w Oct to Feb 2020 but in the methodology under study setting you’ve written that this was b/w Nov to Feb 2020. Please clarify.

**Response**

**Thanks for the comment. This study started on November 2019 and ended in Febraury 2020. The study took sixteen weeks, not six weeks ……Lines 8, pages 2 and lines 85-86, pages 5**

**Comment # 03, Reviewer 1:** You’ve also indicated that the survey was conducted over six weeks, yet the two time periods you have written down are far longer than this. Please clarify.

**Response**

**We conducted concurrently two surveys using the same questionnaire. In particular this study was conducted for sixteen weeks from November 2019 to Febraury 2020 whereas the second survey on antimicrobial stewardship practices started in October 2019 and ended in Febraury 2020**

**Comment # 04, Reviewer 1 :** •The sampling process you have described seems to be more along the lines of a purposive sample comprising three cohorts as opposed to the three staged sampling you have stated in your manuscript. The fact that you have conditionally selected hospitals based on proximity and referral shows that your sample is unlikely to have been truly random. Further, it would have been impractical to randomly select three hospitals out of four as indicated in the third cohort in your sample. Overall, you will need to think hard on the sampling process and describe this appropriately.

**Response**

**We have improved the writing to improve the clarity of our sampling procedure……Lines 114-120, pages 7**

**Comment # 05, Reviewer 1:** •Your thought appears to have trailed off in lines 122- 124 as the sentence is incomplete. Please review

**Response**

**Thanks for the correction. We have completed the sentences**

**Comment # 06, Reviewer 1 :** •In lines 130 – 132, you mentioned you did not provide a definition of what constitutes an ASP. I therefore wonder how ASP was operationalised in the study to ensure that your respondents clearly understood what this was.

**Response**

**Thanks for the observation. The definition of antimicrobial stewardship programme was provided at the moment when the study sought consent before the participant was interviewed, we gave a clear background of what ASP mean and their characteristics (S2_informed consent)**

**Comment # 07, Reviewer 1:** •Lines 149 – 152, please state the number of experts and workshops involved in the development of your questionnaire.

**Response**

**We have added the number of experts who were involved in the development of the questionnaire…………………Lines 148-151,pages 8**

**Comment # 08, Reviewer 1 :** •Lines 176 – 177, how many questionnaires were discarded using the process you have described? Please provide full details.
**Response**

**There was no questionnaire discarded during the process of development of the questionnaire.**

**Over three drafts were reviewed where every question for each section generated was discussed in detail till a one day workshop was suggested to review in detail the three section**

**The fifth draft of the questionnaire was reviewed under the one day workshop. The experts agreed on the readability, clarity, and comprehensiveness of the questions on the three sections ASP, AMS strategies and challenges of AMS implementation.**

**After reviewing the corrections suggested from the workshop, the sixth draft of the questionnaire was recommended by the chair of the workshop for pilot testing. The questionnaire was pre-tested among twenty healthcare providers who were members of the infection control committee to check for clarity and comprehension.**

**The pilot testing of questionnaires generated feedback on ambiguous, misleading and confusing question items. We asked participants what questions needed reformulating and rewording and which ones needed removing because of repetition.**

**The feedback from the pilot testing was shared in a meeting with the ten experts. The meeting resolved all the queries raised by the pilot, which generated a seventh draft. The final questionnaire was approved after testing reliability using the Cronbach alpha to be used in this study**

**Information gotten from literature search was used to develop the first draft which underwent validation of face and content before pilot testing. Information from pilot testing was used to improve the tool.**

**Comment # 08, Reviewer 1:**3. Results. No comments

**Comment # 09, Reviewer 1:** 4. discussion
please do not quote and report your study results in this section as this will be repetitive

**Response**

**We have improved this in our discussion section, Lines 267, pages 22**

**Comment # 010, Reviewer 1:** 5. Conclusions. No comments
**Response**

**Thanks for the comments**

**Comment # 010, Reviewer 1:** Best wishes.

**Thanks so much for the valuable time and comments**

Reviewer #2: There are a number of ambiguities and grammatical errors in the manuscript which need to be addressed. I have added comments and highlighted the relevant sections in the manuscripts for the authors' attention. The manuscript with enclosed comments will be uploaded.

6. PLOS authors have the option to publish the peer review history of their article ([what does this mean?](https://journals.plos.org/plosone/s/editorial-and-peer-review-process#loc-peer-review-history)). If published, this will include your full peer review and any attached files.

If you choose “no”, your identity will remain anonymous but your review may still be made public.

**Do you want your identity to be public for this peer review?** For information about this choice, including consent withdrawal, please see our [Privacy Policy](https://www.plos.org/privacy-policy).

Reviewer #1: No

Reviewer #2: No

[NOTE: If reviewer comments were submitted as an attachment file, they will be attached to this email and accessible via the submission site. Please log into your account, locate the manuscript record, and check for the action link "View Attachments". If this link does not appear, there are no attachment files.]

While revising your submission, please upload your figure files to the Preflight Analysis and Conversion Engine (PACE) digital diagnostic tool, <https://pacev2.apexcovantage.com/>. PACE helps ensure that figures meet PLOS requirements. To use PACE, you must first register as a user. Registration is free. Then, login and navigate to the UPLOAD tab, where you will find detailed instructions on how to use the tool. If you encounter any issues or have any questions when using PACE, please email PLOS at [figures@plos.org](mailto:figures@plos.org). Please note that Supporting Information files do not need this step.

**Reviewer 2**

**Comment # 01, Reviewer 2 :** Authors should consider reviewing abstract and remove repetitions. word limit is 300, they have 317. That can be reduced if repetition are removed

**Response**

**After the removal of repetitions, the abstract is less than 300 words**

**Comment # 02, Reviewer 2 :** In our study, we assessed the characteristics of ASPs, implemented AMS strategies and the challenges to their implementation in hospitals in Uganda.
Consider rephrasing as follows: 'This study assessed....'

**Response**

**Thanks for the comment. We have corrected this……..Lines 4-5, pages 2**

**Comment # 03, Reviewer 2 :** AMS has not been defined in the preceding sentences. Should be defined/written in full at first mention

**Response**

**Thanks for the comment. We have corrected Line 5, page 2**

**Comment # 04, Reviewer 2:** An interview administered questionnaire was used to collect data on the presence of ASP in the studied hospitals, the characteristics of ASP, the implementation of the AMS strategies, and the challenges of implementing ASP.

**Response**

**Thanks for the comment. The implementation of the AMS strategies has been changed to AMS strategies implemented………………… lines 10 pages 2**

**Comment # 05, Reviewer 2:** We analysed all data using descriptive statistics……Delete “all”

**Response**

**We have corrected……………………….Lines 11 pages 2**

**Comment # 06, Reviewer 2**
A total of 32 heads of infection prevention committees of the study hospitals were enrolled on the study…Repetitive of study hospital.

**Response**

**We have deleted the repetition “of the study”………Lines 11 to 12, pages 2**

**Comment # 07, Reviewer 2:** Of these, eight (08) were 12 from regional referral hospitals,……….Delete (08)

**Response**

**We have deleted the figure “(08)” and left it in words………………… Lines 12, pages 2**

**Comment # 08, Reviewer 2:** twenty-one (21) were from general hospitals, and three (03) were from private-not-for profit (PNFPs)…….. Delete twenty-one

**Response**

**We have deleted twenty-one in words and maintained in its figures…………………. Lines 12 to 13, pages 2**

**Comment # 09, Reviewer 2:** twenty-one (21) were from the general hospitals, and three (03) were from private-not-for profit (PNFPs)…….. Delete 03

**Response**

**We have deleted figure “03” and maintained it words………………………….Lines 13 pages 2**

**Comment # 10, Reviewer 2:** Of these, eight (08) were from regional referral hospitals, twenty-one (21) were from the general hospitals, and three (03) were from private-not-for profit (PNFPs)……… PNFPs has been defined; write the abbreviation in subsequent sentences.

**Response**

**We have corrected PNFPs ………………………..Lines 12 to 13, pages 2**

**Comment # 11, Reviewer 2 :** Sixty-eight percent (22/32) hospitals implemented pre-authorisation and approval as their primary AMS core strategy in optimising antibacterial use. ………………Missing word: 'of the'

**Response**

**We have added the words “of the”…………Lines 17, Pages 2**

**Comment # 12, Reviewer 2:** Sixty-eight percent (22/32) hospitals implemented pre-authorisation and approval as their primary AMS core strategy in optimising antibacterial use……………'to optimise' not 'in optimising'

**Response**

**We have corrected as commented …………………….Lines 18, pages 2**

**Comment # 13, Reviewer 2 :** The article 'a' in this sentence is not necessary, and is repetitive. Consider rephrasing to: '... lack of time and allocated funds...'

**Response**

**We have corrected the article “a”……………….Lines 20, pages 2**

**Comment # 14, Reviewer 2:** Can the authors use 'comma', instead of using 'and' three time in one sentence?

**Response**

**We have corrected the statement with commas……….lines 30 to 31, pages 3**

**Comment # 15, Reviewer 2 :** You have just defined 'AMS' as antimicrobial stewardship in the preceding sentence, no need writing the term and abbreviation a second time in the next sentence. Use abbreviation in subsequent sentences.

**Response**

**Thanks for the correction, we have made the corrections………………..Lines 33 to 34, pages 3**

**Comment # 16, Reviewer 2 :** The IDSA categorise AMS into two: core & supplemental strategies. Core strategies are two, not three:a) formulary restriction & preauthorisation (is one strategy, not two) b) prospective audit/review with intervention & feedback. Consider reviewing.

**Response**

**We have reviewed the statement as stated in Lines 40 to 42, pages 3**

**Comment # 17, Reviewer 2 :** Authors should consider reviewing this sentence.

**Response**

**We have reviewed the statement as stated in Lines 49 to 50, pages 3**

**Comment # 18, Reviewer 2:** Delete intervention, rather say, 'AMS strategies implemented....'

**Response**

**Thanks for the comment. We have corrected the statement …………….Lines 52 , pages 4**

**Comment # 19, Reviewer 2 :** ASP has previously been defined in first sentence of introduction and abbreviation used in subsequent sentences. Be consistent with use of abbreviation, no need defining in this sentence again.

**Response**

**Thanks for the correction. We have improved the consistence of the abbreviation……….Lines 77, pages 6**

**Comment # 20, Reviewer 2 :** Has already been defined, use abbreviation

**Response**

**We have corrected ………….Lines 85 to 86, pages 5**

**Comment # 21, Reviewer 2 :** Do you need write the term and abbreviation always?

**Response**

**We have noted this ………. Lines 87 to 88, pages 5**

**Comment # 22, Reviewer 2 :** Review this sentence.

**Response**

**We have improved the clarity of the sentence after rewriting it……………………Lines 88-89, Page 5**

**Comment # 23, Reviewer 2 :** health? Review the sentence

**Response**

**We have corrected the sentence by removing the word “health”………Lines 97, page 6**

**Comment # 24, Reviewer 2 :** This should be 'implement' not 'implementing'

**Response**

**We have corrected the statement by adding the word “implement’…………..Lines 101, pages 6**

**Comment # 25, Reviewer 2 :** Already defined. Use abbreviation

**Response**

**We have corrected the abbreviation…………………………….Lines 106 , pages 6**

**Comment # 26, Reviewer 2:** regional referral hospital? 'hospital' is missing

**Response**

**We thank you for the comment, we have added the word hospital**

**Comment # 27, Reviewer 2 :** Review this whole sentence.

**Response**

**We have improved the clarity of the statement …………………..Lines 127-128, pages 7**

**Comment # 28, Reviewer 2 :** Consider using 'in addition to' rather than 'on top of'

**Response**

**We have improved the writing by replacing 'on top of' with in addition to…………………….Lines 144, pages 8**

**Comment # 29, Reviewer 2 :** Why is 's' in uppercase here?

**Response**

**Thanks for the correction. The capital S in the sentence has been corrected……………………Lines 145, pages 8.**

**Comment # 30, Reviewer 2 :** Review this sentence

**Response**

**Thanks for the comment, We have improved the clarity of the statement ………………..Lines 149-150 ,Pages 8**

**Comment # 31, Reviewer 2 :** 'pre-testing' or 'pre-tested.'? Check the grammar

**Response**

**We have corrected the sentence……………………Lines 161, pages 9**

**Comment # 32, Reviewer 2 :** repetition

**Response**

**We have corrected and removed the repetition………………………..Lines 178-179, Pages 10**

**Results**

**Comment # 33, Reviewer 2:** Which are the two AMS policy strategies?

**Response**

**We have improved the clarity of statement by adding the two AMS policy strategies ……………..Lines 226-227, pages**

**Comment # 34, Reviewer 2 :** See comment in lines 40 & 41.

**Response**

**We harmonised to two core AMS strategies in the write up by removing restriction of formulary restriction ………Lines 229 to 230, pages 14**

**Comment # 35, Reviewer 2**

Repetition.

**Comment # 36, Reviewer 2 :** Repetition. Two 'performed' in one sentence.

**Response**

**We have corrected the repetition…………….Lines 239, pages 17**

**Comment # 37, Reviewer 2 :** In your opening sentence in this section, you reported lack of time and funds were the most reported challenges. The next sentence says lack of education & training was the most reported. Which is corrected?

**Response**

**We have corrected the statement by only maintaining the most commonly reported challenges to the implementation of ASP  across all 32 hospitals (with or without ASP) that is a lack of time for the ASP team and a lack of allocated funding for the antimicrobial stewardship team.**

**Comment # 38, Reviewer 2 :** How do explain the presence of AMS team in a hospital that has not implemented ASP?

**Response**

**In most hospitals studied, medicines and therapeutics committees were present with antimicrobial stewardship subcommittees but the programmes were not established ……………………..Lines 250-251, pages 17.**

**Comment # 39, Reviewer 2:** How is this different from lack of time for AMS team?

**Response**

**Funding and time in relationship to ASP team are different in that in most hospitals funding is required to facilitate AMS strategies and activities while a lack of time is related to the few staff in the hospitals who get so busy with patient and lack limited time for conducting antimicrobial stewardship activities.**

**Comment # 40, Reviewer 2 :** Consider reviewing these sentences. They are ambiguous and do not show a link with the preceding sentence.

**Response**

**We appreciate the comment. We have improved the clarity of the our writing ……………………….Lines 298-301, pages 22**

**Comment # 41, Reviewer 2 :** WHO has already been defined. Stay consistent with abbreviation.

**Response**

**Thanks for the comment. We have corrected the abbreviation of “WHO.”……………….Lines 318 , pages 23**

**Comment # 42, Reviewer 2 :** Ambiguous, review the sentence.Could read like: ' we used only one head or chair of infection control committee, thus we could not verify...'

**Response**

**We have improved the clarity of the statement………………. Lines 328-329, pages 24**

**Comment # 43, Reviewer 2**

However, the position of head of the infection control committee and their active involvement in hospital activities may have regulated their overestimation or underestimation of responses about the ASP…………….. How?

**Response**

**By the study selecting Heads or chairs of IPC, we selected a group of persons who were highly informed on Antimicrobial stewardship. Heads of IPC are members of several hospital administration committees, keeping them informed on hospital matters, including antimicrobial stewardship.**

**Comment # 44, Reviewer 2 :** What do you mean by ASP coverage? Ambiguous and confusing statement. Consider reviewing.

**Response**

**We have reviewed this statement, we have replaced coverage with implemented …………………………Lines 249 ,pages 21**
